# Detection and diagnosis of the early caries lesion

**DOI:** 10.1186/1472-6831-15-S1-S3

**Published:** 2015-09-15

**Authors:** J Gomez

**Affiliations:** 1 Dental Health Unit, School of Dentistry, University of Manchester, Manchester, UK

## Abstract

The purpose of this manuscript is to discuss the current available methods to detect early lesions amenable to prevention. The current evidence-based caries understanding, based on biological concepts, involves new approaches in caries detection, assessment, and management that should include non-cavitated lesions.

Even though the importance of management of non-cavitated (NC) lesions has been recognized since the early 1900s, dental caries have been traditionally detected at the cavitation stage, and their management has focused strongly on operative treatment. Methods of detection of early carious lesions have received significant research attention over the last 20 years. The most common method of caries detection is visual-tactile. Other non-invasive techniques for detection of early caries have been developed and investigated such as Quantitative Light-induced Fluorescence (QLF), DIAGNOdent (DD), Fibre-optic Transillumination (FOTI) and Electrical Conductance (EC). Based on previous systematic reviews, the diagnosis of NCCLs might be more accurately achieved in combination of the visual method and the use of other methods such as electrical methods and QLF for monitoring purposes.

## Introduction

Dental caries is the most prevalent chronic disease worldwide. When initial lesions are taken into account in clinical assessment, only a few individuals are truly unaffected. In most industrialized countries 60-90% of school-aged children are affected and nearly 100% of the adult population is affected [1]. However, in recent years, the patterns of disease presentation have changed. The progression of non-cavitated lesions seems to be slower [2], allowing preventive strategies to be implemented when the lesions have the greatest opportunity to arrest. Traditional methods combined with more sensitive methods may improve the caries diagnosis and also help the clinician in monitoring non-operative treatments. Also, clinical trials involving thousands of subjects and for long periods of time are unrealistic today and the use of cavitated endpoints questionable [3].

Clinical caries measures involving “pre-cavitation” lesions have in fact been reported in caries clinical trials since 1965 [4] and have been described and used in clinical research and practice for more than 50 years [5]. However, some approaches still used in dental practice and in clinical trials have focused on detecting lesions at a cavitation stage informing only restorative decisions [6].

Several conferences have also been held during the past years focused on caries detection and management. In the last Consensus on Diagnosis and Management of Dental Caries, the inability to accurately identify early caries lesions and the need for a change in the system with respect to the non-surgical management of non-cavitated lesions was highlighted [7]. The Consensus Panel concluded the evidence-base for current methods of detection and activity assessment of non-cavitated lesions was not sufficiently strong to recommend their formal adoption [8].

An International Consensus Workshop on Caries Clinical Trials (ICW-CCT) [9] concluded:

- Lesion detection implies an objective method of determining whether or not the disease is present, lesion assessment which aims to characterize it once it has been detected and caries diagnosis which implies a human professional summation of all available data.

- Visual diagnosis is the standard of caries diagnosis; the use of additional methods should be explored further.

- Bitewing radiography adds information to the diagnosis.

- The future of clinical trials, recording only cavitated lesions as an outcome is becoming outmoded.

- Caries measurement methods should accurately capture any signs of the manifestations of the caries process at any given point in time, be able to monitor different levels of de/remineralisation and differentiate product effects in terms of lesion initiation and lesion behaviour (progression, arrest and/or regression).

In spite of all this evidence available, preventive strategies have not been utilized efficiently by the profession. There are a number of reasons for this - perhaps due to failure to observe successful outcomes, financial pressures and the inability to detect lesions at an early stage sufficient for effective prevention. The key problem is that operative care has remained the central management strategy for caries control in general practice, which has negatively impacted on caries epidemiology, clinical outcomes, and patients' quality of life, among others.

The medical model based on early detection of the disease integrates a successful risk assessment based on an understanding of the disease process. Caries risk assessment is one of the cornerstones in patient caries management and should be carried out and documented in the patient's chart either for treatment planning or as a didactic aid for patient motivation [10]. However, the existing evidence on Caries Risk Assessment systems is limited and comes from cross-sectional studies where various multivariate regression techniques were deployed to identify methods for classifying individuals based on their caries risk status [11]. These studies are inadequate for correctly identifying the individuals at risk for caries, which is the determining characteristic of an ideal CRA system. Longitudinal prospective studies, on the other hand, assess the prediction of new caries development, which, with limitation, is stronger than a single assessment of risk factors. Unfortunately, there are few prospective studies of good quality available.

Diagnosis has been defined as “the art or act of identifying a disease from its signs and symptoms” and caries detection is the signs and symptoms identified [12].

There is often confusion in the literature in the terminology used for caries detection and caries diagnosis. In the last decade, three terms have been agreed in terms of direct relevance to preventive caries care: [13]*lesion detection:*implies an objective method of determining whether or not disease is present; [14]* lesion assessment: *aims to characterise or monitor a lesion, once it has been detected, and (3) *caries diagnosis: *should imply a human, professional, summation of all available data [9].

It has been stated that a good detection method should be valid and reliable [6]. A valid method results in measurements compared with a gold standard. In caries, detection performance has been assessed using at 2x2 contingency table containing the distributions of the true positives, true negatives, false positives and false negatives. Sensitivity and specificity are widely used measures to describe and quantify the diagnostic ability of a test [15] and are expressed as values between 0 and 1 (100%), values closer to 1 indicating a high quality result. Those values will depend on the distribution of caries on the studied sample. Often the caries prevalence of the sample studied in the in vitro studies is high (50-90%) compared with real clinical situations, overestimating the sensitivity at disease level. The inclusion of too many sound surfaces in a sample of a study will cause an overestimation of specificity [16]. The variation of the sensitivities and specificities varies depending on the thresholds level. It has been shown that when the detection of the disease is made at the non-cavitated level, the DMF can be doubled and the sound surfaces were decreased to approximately one-quarter [17]. The concept of reliability of a method is also important. A reliable diagnostic is a method that can be used by one or different examiners so they should obtain identical results [6]. Visual diagnosis combined with bitewing radiography is the most common method of caries diagnosis and the use of additional methods, mainly for monitoring purposes should be explored further. A range of new detection systems have been developed and are either currently available to practitioners or will shortly be made so. During the last decade, International Caries Detection and Assessment System (ICDAS) system has been considerably used and submitted to extensive research. The International Caries Detection and Assessment System (ICDAS) was developed in 2001 by an international group of researchers. The system was proposed as a strategy to integrate the modern detection systems into one standard system [18]. The ICDAS incorporate concepts from the research conducted by Ekstrand et al. [19,20], Fyffe et al. [21] and other caries detection systems described in the systematic review conducted by Ismail (2004) [22]. The ICDAS is the subdivision of stages of the continuum of dental caries into a variable number of discrete and predictable categories based upon the histological extent of the lesion within the tooth [23,24]. ICDAS identifies caries lesions on the basis of their clinical visual appearance (Table 1) [18]. The examination is visual aided by a ball-ended explorer and should be carried out on clean and dry teeth [18]. The assessment of lesion activity is also very important when using ICDAS. Lesion activity assessment will help on the treatment decisions, particularly when preventive options should be implemented [25]. ICDAS has shown to be an accurate and reproducible method to detect early lesions and also to detect changes in longitudinal follow-up [26,27].

**Table 1:** ICDAS scores

Score	Criteria
0	Sound

1	First Visual Change in enamel

2	Distinct Visual Change in enamel

3	Localized enamel breakdown

4	Underlying dentine shadow

5	Distinct cavity with visible dentine

6	Extensive cavity with visible dentine

In the past years quantitative methods for detecting and monitoring of carious lesions have been introduced. Some reasons for the development of these methods are: 1) quantitative methods can detect earlier carious lesions than conventional methods, 2) quantitative methods can be more reliable than qualitative methods, and 3) quantitative assessments can monitor the course of the disease [28].

Radiographs are the most used detection aid using the bitewing technique. The aim of the bitewings is to detect proximal caries lesions that cannot be detected in the visual inspection. It has been shown in the literature that the use of radiographs is more sensitive than clinical inspection for detecting approximal lesions and for occlusal lesions in dentin, for estimating depth of the lesion, and for monitoring lesion behavior [8,29]. Furthermore, in occlusal surfaces, the contribution of the radiographs seems to be minimal [30]. When an occlusal lesion is detected on a bitewing radiograph, the lesion may have already reached the middle third of dentine and hence beyond the scope of remineralisation interventions [31]. Moreover, radiography cannot distinguish between active and arrested lesions and sometimes between non-cavitated and cavitated lesions [32]. This last fact should be definitely be determined before undertaking any operative intervention. It has been suggested that temporary tooth separation can offer to clinicians the ability of determining if the lesion is active/inactive, cavitated/non-cavitated [33]. The most common caries detection method is the combination of visual-tactile examination with bitewing radiography. However, some studies have shown the decrease of performance when using the combination of both methods. The accuracy of the visual-tactile examination alone will depend on the method used for this purpose, the use of probes for tactile assessment and the ability to perform tooth separation to detect approximal lesions [34]. One of the criteria to assess proximal caries lesions is the one proposed by ICDAS (Table 2). Radiographic examination should be included as part of the initial patient assessment and also in the process of monitoring lesion behaviour over time. Radiography can add information about the clinical stages of the caries process at approximal surfaces and the more advanced stages on occlusal surfaces [32].

**Table 2:** Scores for radiographical classification of lesion severity

Score	Criteria
0	no radiolucency

1	radiolucency in outer ½ of the enamel

2	radiolucency in inner ½ of the enamel ± EDJ

3	radiolucency limited to the outer 1/3 of dentine

4	radiolucency reaching the middle 1/3 of dentine

5	radiolucency reaching the inner 1/3 of dentine, clinically cavitated

6	radiolucency into the pulp, clinically cavitated

**Figure 1:**
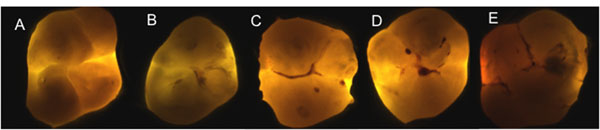
Example of FOTI images. A: No shadow; B: Thin-grey shadow into enamel; C: Wide-grey shadow into enamel; D: Microcavitated lesion shadow <2 mm in dentine; E: Shadow >2 mm in dentine.

Transillumination can also be an useful tool in the detection of approximal caries. Fiber-optic transillumination (FOTI) is based on the phenomenon of light scattering to increase contrast between normal and carious enamel. Sound enamel is comprised of modified hydroxyapatite crystals that are densely packed, ‘producing an almost transparent structure. Dentine appears orange, brown, or grey underneath enamel and this can help in the discrimination between enamel or dentine lesions (Figure 1). DIFOTI replaces the human eye with a CCD sensor. The transillumination method may support a treatment decision-making but it is not capable of monitoring dental caries lesions as the bitewing radiographs [35]. Recent developments in ordinal scales for visual assessments, such as the ICDAS scoring system, may enable a more robust framework for visual exams into which FOTI can be added [36].

ECM is another method proposed for caries detection. Demineralisation, in theory, creates porosities; the porosities will fill with water and ions from saliva causing electrical conductivity changes [37]. The ECM device employs a single, fixed-frequency alternating current, which attempts to measure the ‘bulk resistance’ of tooth tissue [38]. The degree of electrical conductance is dictated by the properties of the substance including porosity, the contact area, the thickness of the tissue, hydration of the enamel, and ionic content of dental fluids [39]. The method has shown promising results showing superior performance to FOTI and radiography in early lesions [29]. However, previous studies have shown the presence of stain as a confounder factor [40]. Another issue is the wide variations on reproducibility, possibly due to inconsistent probe contact with the tooth surface [41]. 

**Figure 2:**
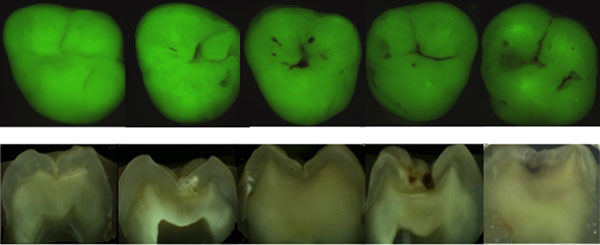
QLF images compared with histological sections.

**Figure 3:**
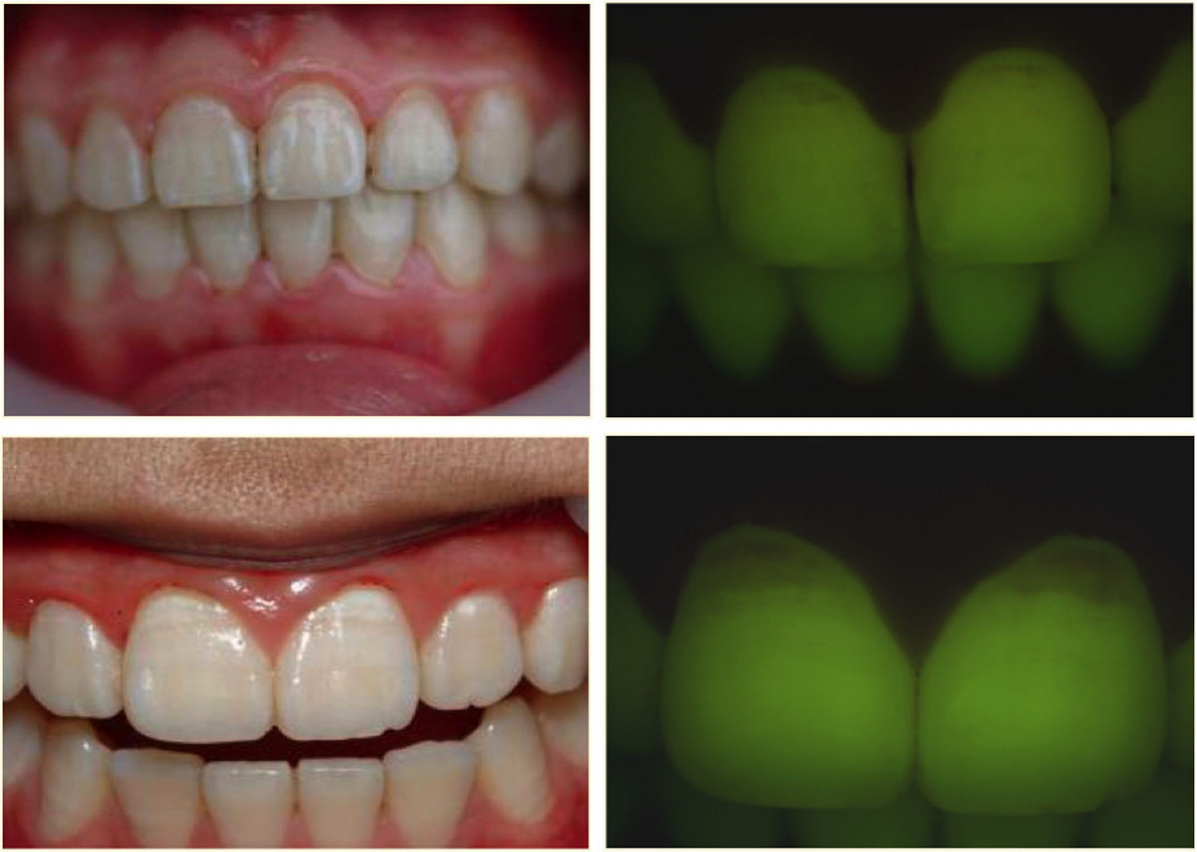
QLF clinical examples

Other methods based on fluorescence have become commercially available in the past years. QLF is a diagnostic aid for detection, quantification and monitoring of early enamel demineralisation. QLF operates on the principle of enamel autofluorescence, detecting and quantifying the loss of fluorescence associated with demineralisation [36]. The technique is based on the principle of the excitation of the dentine with blue light (370 nm) and would make it fluoresce into yellow-green region. When a lesion is present, an increase of light scattering makes the lesion appear as dark spots on a bright green background. The loss of fluorescence images can be quantified with respect to adjacent healthy tissue [42]. The fluorescent image of the tooth is recorded and digitalized and analyzed quantitatively. The loss of fluorescence is obtained by reconstruction of the fluorescence of healthy enamel, assuming that is 100%. The decrease in fluorescence is determined by calculating the percentage difference between the actual and the reconstructed surface. Any area with a decrease in fluorescence over 5% is considered as a lesion [43]. The reliability of the QLF in vivo appears to be excellent for the quantification of initial caries lesions on smooth surfaces [44]. QLF has shown good sensitivity in vivo [27]. However, the specificity is sometimes compromised due to the confounding factors. Correlations of up to 0.82 have also been reported for QLF metrics and lesion depth [36]. QLF has also shown the ability to detect and quantify changes of mineral content and size of lesions by demonstrating a dose response between F and non-F dentifrices in short-term clinical trials [44,45]. QLF is a potential tool for detection of early carious lesions and for the monitoring of preventive interventions. Some examples of in vitro QLF images compared with histological sections and in vivo images are shown in Figures 2 and 3. Another method based upon the imaging and auto-fluorescence of dental tissues to detect caries is the SoproLife® camera [46]. The camera operates in three modes: for daylight mode four white light LEDs are engaged; for the diagnostic and treatment modes the light is provided by four blue LEDs (450 nm). A new camera, the Soprocare®, also provides three clinical modes: daylight, caries and periodontal mode. The literature on SoproLife® is limited to preliminary results only.

Another caries detection device based on fluorescence is DIAGNOdent (DD). The DIAGNOdent device consists of 655 nm monochromatic light that is emitted from a tip/sensor and can detect back-scattered fluorescence from the tooth [47]. At 655 nm, the fluorophores have been identified as bacterial porphyrins. The DD scores range between 0 and 99. This number offers the possibility to monitor lesion behaviour [39]. In previous systematic reviews considering fluorescence methods (LF), a tendency of higher specificity than sensitivity, except for the dentine threshold, was observed. The main issue of low specificities at the dentine level is the over prescription of operative treatment. The performance of LF seems to be better for more advanced lesions [48]. A recent systematic review found a wide variation in design features including the threshold for DD scores, the validation methods and the outcomes expressed, among others [29]. In general, DD evidence seems to be stronger for smooth and occlusal caries detection than for approximal and for permanent dentition than for the primary dentition. Factors that may influence the outcome of the measurements in different ways are the presence of plaque, calculus and/or staining on the tooth surface, and the degree of dehydration of tooth tissue, among others [49]. Hence, there is a poor correlation between LF readings and the mineral content, but possibly better correlation with the presence of infected dentine. Initial lesions are less infected than dentinal lesions [50], which hamper the performance of fluorescence-based methods in detecting such lesions as the method detects the presence of bacterial metabolites. Previous studies suggest that white-spot lesions formed in vitro, without the involvement of bacteria, do not produce a significant increase of fluorescence, although more advanced lesions (D2 and D3) produce a distinctive fluorescence, indicating that DD measures the fluorescence of bacteria or their metabolites [51]. Some care is required in the use of Diagnodent in clinical studies, due to problems with stain- and plaque-confounding assessments, and perhaps further work is required before it can be used routinely in clinical studies. The systematic review of Diagnodent [52] confirms the need for caution in both clinical practice and research use.

Another emerging technology is the Canary Dental Caries Detection System. The technology is based on the detection of optical and thermal changes using a combined t

Dental caries is a reversible disease that can be halted at any given point, as long as the biofilm can be removed. The very early changes in the enamel can be detected with traditional visual-tactile methods; other additional tools can be used for monitoring purposes in practice and in clinical trials. Different stages to classify dental caries have been proposed based on activity, visual signs and extent of the lesions. Additional methods such as radiographs, FOTI, Laser Fluorescence, ECM among others can also be used for monitoring disease, in particular in clinical trials assessing efficacy of anti-caries dental care products.

All caries detection methods are subject to errors with less than perfect reliability and validity [34]. The detection of caries lesions should be focused on the exoneration of sound surfaces, instead on the detection of lesions biased towards the restorative approach. False positive diagnoses are more dangerous in terms of unnecessary invasive treatments [34]. However, dentists are normally more focused on the detection of lesions than on the exoneration of sound surfaces, particularly to avoid overlooking deep lesions. It is at this moment that clinicians tend to use additional methods to complete the decision of when to intervene [34].

In a recent review about caries detection methods, one important source of heterogeneity found in the studies assessing detection systems is the inconsistent using different thresholds. For example, some studies report D1 combining enamel and dentine or other collapsing sound and enamel. The results on NCCLs are inconclusive for some methods and it seems that the diagnosis can be improved in combination of visual assessment such as ICDAS and other quantitative methods [29].

In terms of caries diagnosis, the main objective of patient care should be to classify the lesions according to their biological representation and provide them with the best biologically oriented treatment in order to preserve tooth structure. The biological rationale is that cavitated lesions will require a restoration, whereas non-cavitated active lesions can be controlled with preventive therapies such as plaque control and fluorides. This objective can only be achieved with visual-tactile clinical examination.

Dental caries continues to be one of the most prevalent diseases and a significant burden for health systems. In recent years evidence has shown the limitation of relying on a restorative approach to manage dental caries. The current biological understanding of the caries process has led to the development of new philosophies based on early detection, preventive management and preservation of tooth structure. However, this approach has not always been reflected in dental education and activity profiles of health providers. Clearly, a restorative bias continues to influence how dentistry is practiced today. This approach has been embedded in under- and postgraduate education, licensing, insurance, finances and reimbursement systems and also in public opinion.

The comparison of all the detection methods available can be difficult. Several validation methods, definitions of disease may pose a challenge for the dental practitioner who is trying to define the best care pathway. Well-established and evidence based methods such as visual assessment and radiographs may be supplemented in some cases by other methods such as Diagnodent, ECM or QLF, for monitoring purposes. It has yet to be established whether methods such as QLF and ECM may become a helpful tool in the detection of dental caries in everyday practice.

Additional caries detection methods should be used as an adjunct to clinical decision- making and for caries diagnosis and treatment planning in conjunction with caries risk assessment. None of these methods should be used as a justification for premature restorative intervention.

## Declarations

This article has been published as part of *BMC Health Services Research* Volume 15 Supplement 1, 2015: Proceedings of Prevention in practice - making it happen. The full contents of the supplement are available online at http://www.biomedcentral.com/bmchealthservres/supplements/15/S1. Publication charges for this supplement were funded by Colgate Palmolive.
